# Abdominal Aortocaval Vascular Injury following Routine Lumbar Discectomy

**DOI:** 10.1155/2014/895973

**Published:** 2014-10-07

**Authors:** Matthew Leech, Marc James Whitehouse, Ruta Kontautaite, Mukesh Sharma, Sumant Shanbhag

**Affiliations:** ^1^Department of General Surgery, University Hospital of South Manchester NHS Foundation Trust, Wythenshawe Hospital, Southmoor Road, Wythenshawe, Manchester M23 9LT, UK; ^2^Department of Anaesthesia and Intensive Care, Walsall Manor Hospitals NHS Trust, Walsall, West Midlands WS2 9PS, UK

## Abstract

Vascular complications following spinal surgery are potentially fatal; however, fortunately they are rare. This risk is often focused on the close proximity of the surgical field to retroperitoneal structures. Prompt diagnosis is essential; however, bleeding is often occult, and this may therefore delay management of this condition. Despite previous reports many clinicians may not be aware of this potentially fatal complication. The overall morbidity and mortality may be reduced by prompt diagnosis and treatment. Clinicians must, therefore, have a high degree of suspicion in all patients who undergo spinal surgery. We therefore present a case of a 51-year-old man who sustained an aortocaval injury during a revisional lumbar discectomy. The patient developed refractory hypotension, which deteriorated into PEA arrest. Emergency laparotomy was performed which revealed an aortocaval injury. Immediate primary vascular repair was successfully performed. The patient was resuscitated and made a full recovery.

## 1. Introduction

Lumbar intervertebral disc surgery is a routine procedure in orthopaedic practice. However, iatrogenic injury of major abdominal vessels during lumbar discectomy via a posterior approach, albeit rare, is a life-threatening complication of spinal surgery. Interestingly, the incidence of such injury has remained static over the last 40 years occurring in 0.01–0.05% of cases [1]. Recent reports indicate that vascular injury is only recognised intraoperatively in 36% of cases and that 28% of presentations occur within the first 24 hours following surgery [2]. A major vessel insult is often concealed; therefore haemodynamic instability may be the only sign of a catastrophic bleed. A subacute presentation may manifest as nonspecific lumbar pain, pedal oedema, or high output cardiac failure [3]. Treatment of lacerations consists mainly of primary suturing of the transected vessel. However suturing from within the arterial lumen as well as interposition grafting remains the preferred approach to repairing arteriovenous fistula and pseudoaneurysms. Minimally invasive endovascular techniques have been used increasingly as an alternative treatment modality to conventional repair because of its low morbidity and mortality [2]. However, this is dependent on the availability of tertiary vascular services, which may not be available locally.

Here we report a case of aortocaval injury sustained during revisional L4/L5 posterior lumbar discectomy for lumbar disc herniation. With prompt diagnosis and immediate management we were able to avert a clinical catastrophe. Our aim, therefore, is to educate clinicians of this rare, but potentially fatal complication and to serve as a reminder for anaesthetists/anaesthetic practitioners to maintain a high degree of suspicion for such eventualities and diagnose promptly and treat accordingly.

## 2. Case Presentation

A 51-year-old man underwent revisional lumbar discectomy surgery for L4/L5 disc herniation. This was performed by an experienced orthopaedic spinal surgeon at our hospital. Prior to induction, intravenous access was established with 20 g cannula to serve for drug and fluid administration. Standard routine monitoring with continuous ECG, pulse oximetry and intermittent noninvasive blood pressure monitoring were recorded and were within normal limits. General anaesthesia with fentanyl, propofol, and atracurium was administered and intubation with a size 8.0 cuffed endotracheal tube was inserted uneventfully. Our patient was placed in the prone position for the procedure on a Relton-Hall frame and anaesthesia was maintained with desflurane, maintaining a minimum alveolar concentration (MAC) of 1.0. Additional doses of atracurium were administered to maintain paralysis and aid in intermittent positive pressure ventilation (IPPV).

The patient underwent a posterior L4/L5 lumbar discectomy. Surgery continued unabated for approximately 2 hours with no anaesthetic or surgical concerns. However, during final closure, our patient became progressively hypotensive and tachycardia with sequential reduction in end tidal carbon dioxide levels. Despite aggressive fluid management and inotropic boluses with ephedrine (21 mg cumulative total) and metaraminol (10 mg cumulative total) his condition continued to deteriorate. On further exploration there was no overt blood loss from the surgical wound or inserted drains. The patient was repositioned into a supine position, where it was instantly apparent that our patient had had an acute intra-abdominal event. He had a grossly distended abdomen with extensive lower limb mottling. An intra-abdominal vascular injury was highly suspected. A vascular surgeon was summoned immediately; however, prior to the arrival of a vascular surgeon, our patient developed a PEA arrest. Advanced life support (ALS) was commenced immediately. Further intravenous access proved difficult; however, intraosseous cannulation was established. Intermittent adrenaline boluses were administered (3 mg cumulative total); 2 units of uncross matched O-negative blood were administered initially, followed by 10 units of type-specific blood; 5 units of FFP and 1 g of cryoprecipitate in total. During resuscitation an emergency laparotomy was performed. The aorta was clamped approximately 14 minutes into the arrest. The vascular surgeon removed a large retroperitoneal haematoma and was able to quickly identify a transected aorta and inferior vena cava perforation. This was repaired successfully with primary closure. ROSC occurred approximately 22 minutes into the arrest. Central venous (right internal jugular vein) and arterial (left axillary artery) access were established. Once stabilised our patient was transferred to intensive care for cardiorespiratory support. Surprisingly, he remained cardiovascularly stable after arrest and did not require any further inotropic support. A chest-drain was inserted due to the development of a pneumothorax secondary to external chest compressions performed during resuscitation. Our patient was ventilated for 24 hours and successfully extubated uneventfully. Fortunately, our patient made a full recovery with no demonstrable neurological deficit as a result of the arrest. He was discharged home six days after his initial procedure.

## 3. Discussion

Major vessel injury during intervertebral disc surgery is a rare but catastrophic complication. There are a multitude of vascular injuries that have been reported in the literature, but typically these include vessel wall laceration, arteriovenous fistula, and pseudoaneurysm formation. Other structures may be compromised and these have included visceral and ureteral damage [4]. These injuries occur due to the proximity of the anterior longitudinal ligament and major retroperitoneal structures located anteriorly. The common iliac artery followed by the common iliac vein is most frequently affected due to its anatomical position immediately anterior to the L4-L5 intervertebral disc space [5]. In case of unexpected circulatory instability during spinal surgery, iatrogenic vascular damage should always be suspected. Prompt diagnosis and immediate intervention can prove lifesaving. Bifurcation of the aorta and inferior vena cava occurs at the level of L4 vertebrae. The left common iliac is most commonly damaged due to its more medial course and close interrelationship with L4-L5 intervertebral disc space, separated only by the anterior longitudinal ligament. Specifically the site of injury occurs in relation to the level of discectomy, with the aorta and vena cava more commonly injured, rather than visceral structures during lumbar discectomies. Chronic degenerative vertebral disease combined with previous surgery may weaken the anterior longitudinal ligament and alter grossly the anatomical field.

Spinal experts agree that arterial injuries (in the majority of cases) are the result of malpositioning of the posterior rongeur. It is thought that injuries occur during intervertebral disc fragment removal and are the result of the posterior rongeur inadvertently penetrating the anterior longitudinal ligament. The prone position ensures that the abdominal structures become pressed against the vertebral bodies rendering the retroperitoneal vessels fixed in close proximity to the anterior longitudinal ligament and thus are at risk of potential impingement and damage by the posterior rongeur. Severe arterial injury of the major abdominal vessels such as in this case can quickly result in life-threatening retroperitoneal haemorrhage.

Catastrophic haemorrhage during lumbar spine surgery is often concealed leading to a crucial delay in diagnosis. This is attributed to haemorrhagic blood flow into the retroperitoneal space rather than into the surgical field and also due to the durable self-sealing effect of the anterior longitudinal ligament [6]. As a result cardiovascular collapse may be the first demonstrable sign of a major vascular injury. Clinicians are therefore reminded to consider a vascular injury in all patients with hypotension, tachycardia, and abdominal distention, particularly in those cases who appear refractory to medical management. Postoperatively, clinicians are reminded to remain vigilant for the first 72 hours since, during this time period, arteriovenous fistula or pseudoaneurysm often develops.

In our case major vessel injury was not suspected until the end of the procedure when the patient was placed into the supine position since the surgical field remained dry. After reviewing the available literature, our experience appears consistent with other similar cases. Furthermore, the period of time elapsed between iatrogenic injury and diagnosis varies significantly from minutes to weeks [7]. Accordingly treatment will vary depending on the nature and severity of the injury. This may vary from primary closure and end-to-end anastomosis to angiographic intervention or graft interposition [8]. In cases of major haemorrhage, as in our case, immediate laparotomy with primary closure is necessary to avert a catastrophe.

## 4. Conclusion

Lumbar intervertebral disc surgery is a routine procedure in orthopaedic practice. Iatrogenic injury of major abdominal vessels during lumbar discectomy is a life-threatening complication of spinal surgery. In our case, the patient sustained an aortic transection and caval perforation, which resulted in cardiovascular collapse. Following prompt resuscitation and an emergency laparotomy with primary vascular closure, our patient survived. This case should, therefore, be learned as a reminder for clinicians to remain vigilant during the perioperative, recovery, and rehabilitation phases.
